# Effects of right stellate ganglion block combined with topical anesthesia on heart rate variability in awake patients receiving tracheal intubation

**DOI:** 10.4314/ahs.v23i2.43

**Published:** 2023-06

**Authors:** Liping Bai, Weihong Hao, Chunmin Zhang, Zhiming Guan

**Affiliations:** 1 Department of Anesthesiology, Shanxi Bethune Hospital, Taiyuan 030032, Shanxi Province, China; 2 Department of Respiratory Medicine, Second Hospital of Shanxi Medical University, Taiyuan 030001, Shanxi Province, China

**Keywords:** Anesthetics, topical, block, heart rate, intubation, intratracheal, stellate ganglion

## Abstract

**Objective:**

General anesthesia is commonly performed with tracheal intubation. We aimed to evaluate the effects of right stellate ganglion block combined with topical anesthesia on the heart rate variability in awake patients receiving tracheal intubation.

**Methods:**

A total of 120 eligible patients were equally divided into observation and control groups using a random number table. The observation group underwent right stellate ganglion block combined with topical anesthesia. The excellent and good rate of anesthesia, indicators of vital signs and heart rate variability, incidence rate of adverse reactions, success rate and time consumption of tracheal intubation, postoperative resuscitation and cognitive function score of the two groups were compared.

**Results:**

The systolic pressure, diastolic pressure and heart rate significantly increased in both groups during tracheal intubation compared with those before tracheal intubation, which they were lower in the observation group than those in the control group during tracheal intubation (P<0.05). Low-frequency power, ratio of low-frequency power to high-frequency power and heart rate variability index significantly decreased in both groups during tracheal intubation compared with those before tracheal intubation (P<0.05).

**Conclusion:**

Right stellate ganglion block combined with topical anesthesia can work well for awake patients during tracheal intubation.

## Introduction

General anesthesia is commonly conducted through tracheal intubation, with satisfactory anesthetic effects^1^-^3^. However, patients are prone to stress responses such as elevated blood pressure and increased heart rate when receiving tracheal intubation in the waking state, thus affecting the success rate of intubation^4^-^6^. Therefore, it is necessary to perform anesthesia management for patients before tracheal intubation.

The stellate ganglion, which is irregularly star-shaped, renders block challenging due to critical structures in the vicinity and potential collateral damage during the operation^7^. Preoperative right stellate ganglion block has been verified to effectively decrease the incidence rate of intraoperative or postoperative atrial fibrillation in patients receiving lung lobectomy^8^. Besides, topical anesthesia is able to effectively suppress tracheal intubation-induced strong stress response in the throat and trachea^9^. Nevertheless, right stellate ganglion block has seldom been combined with topical anesthesia for tracheal intubation awake hitherto.

In this study, therefore, right stellate ganglion block combined with topical anesthesia was performed for patients in the waking state, and then tracheal intubation was implemented, aiming to assess the effects on the heart rate variability and to provide valuable evidence for future clinical treatment.

## Materials and methods

### General data

A total of 120 patients receiving general anesthesia with tracheal intubation in the waking state in our hospital from January 2019 to October 2020 were enrolled and equally divided into two groups using a random number table. There were 33 men and 27 women aged 20-59 years old, with an average age of (39.89±9.02) years old, in control group. Observation group consisted of 32 males and 28 females with an age of (19±-59) years old and an average of (39.76±9.12) years old. There were no significant differences in the gender and age between the two groups (P>0.05), which were comparable. This research was reviewed and approved by the Medical Ethics Committee of the hospital, and the patients and their families signed the informed consent.

### Methods

All patients were fasted for 8 h and deprived of water for 4 h before operation. After entering the operating room, a venous access was built, 10 mL/kg Sodium Lactated Ringer's Injection was infused intravenously, and the patients were connected to an electrocardiogram monitor to supervise the heart rate and blood pressure.

Simple topical anesthesia was applied in control group. Specifically, 1.33% lidocaine was mixed with 1% ephedrine, and the mixture was applied to the nasal mucosa and contracting nasal mucosa for topical anesthesia using cotton swabs. Later, through thyrocricoid puncture, 2 mL of 2% lidocaine was injected into the trachea to anesthetize the surface of the throat.

Right stellate ganglion block combined with topical anesthesia was adopted in observation group. Specifically, 1.33% lidocaine was mixed with 1% ephedrine, and the mixture was applied to the nasal mucosa and contracting nasal mucosa for topical anesthesia using cotton swabs. In the supine position without pillow, the patients were punctured by a needle inserted vertically at 1.5 cm lateral to the horizontal midline of cricoid cartilage and 2.5 cm above the sternoclavicular joint. Then the puncture needle was retreated by 2 mm when touching the bone, and no cerebrospinal fluid and blood were withdrawn. Next, 6 mL of 1% lidocaine was injected into the stellate ganglion. Finally, through thyrocricoid puncture, the surface of the throat was anesthetized by injecting 2 mL of 2% lidocaine into the trachea.

The patients in both groups were further subjected to intravenous injection of 1 µg/kg fentanyl and 20 µg/kg midazolam, and nasotracheal intubation was performed under the guidance of fiberoptic bronchoscope 3 min later. The tracheal intubation was manipulated by the same anesthesiologist.

### Observation indices

The excellent and good rate of anesthesia, vital sign indicators (including systolic pressure, diastolic pressure and heart rate), indicators of heart rate variability (e.g. low-frequency power, ratio of low-frequency power to high-frequency power and heart rate variability index), incidence rate of adverse reactions of tracheal intubation, success rate of tracheal intubation, time consumption of tracheal intubation, postoperative resuscitation (such as recovery time of spontaneous respiration, time to eye opening and time of response recovery to command after operation) and cognitive function score were compared between the two groups.

The anesthetic effect was evaluated based on the conditions of patients during tracheal intubation in accordance with the following criteria: 1) excellent: Patients kept quiet, without pain, coughing and restlessness. 2) Good: Patients had mild pain within tolerance range and with no impact on intubation. 3) Poor: Patients had obvious pain, coughing and restlessness that forcedly interrupted the tracheal intubation. Excellent and good rate = excellent rate + good rate.

The cognitive function was assessed using Montreal Cognitive Assessment (MoCA) scale and Mini-Mental State Examination (MMSE) scale. The MoCA scale was scored 0-30 points in total, which was directly proportional to the cognitive function. With 26 points as the critical value, the score <26 points indicated cognitive impairment, and the higher the score was, the better the cognitive function would be. The total score of the MMSE scale was 0-30 points and directly proportional to the cognitive function. With 17 points as the critical value, the score <17 points meant cognitive impairment, and a higher score signified better cognitive function.

### Statistical analysis

SPSS 22.0 software (IBM Inc., USA) was employed for statistical analysis. The numerical data were expressed as n and examined by the χ^2^ test, and the measurement data were represented as (*x̅ ± s*) and analysed using the t-test. P<0.05 suggested that a difference was statistically significant.

## Results

### Anesthesia excellent rate

The anesthesia excellent rate was 98.33% in observation group, which was higher than that (86.67%) in control group (P<0.05) (Table 1).

**Table 1:** Anesthesia excellent rate [n (%)]

Group	n	Excellent	Good	Poor	Excellent and good rate
Control	60	31 (51.67%)	21 (35.00%)	8 (13.33%)	52 (86.67%)
Observation	60	36 (60.00%)	23 (38.33%)	1 (1.67%)	59 (98.33%)*

* P<0.05 *vs*. control group.

### Vital sign indicators

The systolic pressure, diastolic pressure and heart rate were significantly increased in both groups during tracheal intubation compared with those before tracheal intubation (P<0.05), while they were lower in observation group than those in control group during tracheal intubation (P<0.05) (Table 2).

**Table 2:** Vital sign indicators (*x̅±s*)

Group	Time	Systolic pressure (mmHg)	Diastolic pressure (mmHg)	Heart rate (beat/min)
Control (n=60)	Before tracheal intubation	106.42±2.73	76.51±1.21	71.24±1.28
	During tracheal intubation	110.93±3.14^#^	79.87±1.78^#^	74.58±1.87^#^
Observation (n=60)	Before tracheal intubation	106.31±2.76	76.43±1.19	71.15±1.30
	During tracheal intubation	106.85±2.91^#^*	78.02±1.45^#^*	72.70±1.45^#^*

# P<0.05 *vs*. before tracheal intubation within the group

* P<0.05 *vs*. control group

### Indicators of heart rate variability

The low-frequency power, ratio of low-frequency power to high-frequency power and heart rate variability index were significantly decreased in both groups during tracheal intubation in contrast with those before tracheal intubation (P<0.05), but observation group had higher levels of those indicators than control group during tracheal intubation (P<0.05) (Table 3).

**Table 3:** Indicators of heart rate variability (*x̅±s*)

Group	Time	Low-frequency power (ms^2^/Hz)	Ratio of low-frequency power to high-frequency power	Heart rate variability index
Control (n=60)	Before tracheal intubation	1647.81±88.52	1.43±0.20	56.74±3.61
	During tracheal intubation	1528.46±43.79^#^	1.10±0.15^#^	50.43±2.38^#^
Observation	Before tracheal intubation	1649.53±89.45	1.44±0.19	56.89±3.50
(n=60)	During tracheal intubation	1584.76±50.13^#^*	1.26±0.16^#^*	53.21±2.74^#^*

# P<0.05 *vs*. before tracheal intubation within the group

* P<0.05 *vs*. control group

### Incidence rate of adverse reactions of tracheal intubation, success rate of tracheal intubation and time consumption of tracheal intubation

Observation group exhibited a lower incidence rate of adverse reactions of tracheal intubation (P<0.05), a higher success rate of tracheal intubation (P<0.05) and less time consumption of tracheal intubation (P<0.05) than control group (Table 4).

**Table 4:** Incidence rate of adverse reactions of tracheal intubation, success rate of tracheal intubation and time consumption of tracheal intubation

Group	n	Coughing	Restlessness	Success rate of tracheal intubation	Time consumption of tracheal intubation (s)
Control	60	10 (16.67%)	8 (13.33%)	55 (91.67%)	43.76±9.41
Observation	60	2 (3.33%) *	1 (1.67%) *	60 (100.00%) *	30.12±8.25*

* P<0.05 *vs*. control group.

### Postoperative resuscitation

There were no significant differences in recovery time of spontaneous respiration, time to eye opening and time of response recovery to command after operation between observation group and control group (P>0.05) (Table 5).

**Table 5:** Postoperative resuscitation (*x̅±s*, min)

Group	Recovery time of spontaneous respiration	Time to eye opening	Time of response recovery to command
Control (n=60)	7.85±0.79	9.18±1.57	17.42±3.07
Observation (n=60)	7.89±0.82	9.23±1.54	17.57±3.20

### Cognitive function scores

The MMSE score and MoCA score were not significantly changed after operation in comparison with those before operation in the two groups (P>0.05), and they were not significantly different in terms of inter-group comparison before and after operation (P>0.05) (Table 6).

**Table 6:** Cognitive function scores (*x̅±s*, point)

Group	Time	MMSE score	MoCA score
Control (n=60)	Before operation	26.51±2.92	24.73±3.15
	After operation	26.27±2.71	24.56±3.06
Observation (n=60)	Before operation	26.42±2.83	24.89±3.27
	After operation	26.20±2.54	24.65±3.11

## Discussion

General anesthesia with tracheal intubation is one of the most commonly used anesthetic methods for surgery, during which an anesthesia ventilator connected with an endotracheal tube can be applied to supply oxygen for patients in the sleep state after anesthesia, so as to maintain oxygen supply to the body, alleviate stress responses during operation and minimize the influence of operative procedures on the body^10^-^12^. However, as the tracheal intubation is invasive, patients tend to have stress responses due to the stimulation of the tube inserted into the trachea. Besides, the sympathetic activity is enhanced, thereby causing difficult intubation^13^-^15^. Hence, it is essential to do a good job in anesthesia before tracheal intubation.

In previous clinical practices, topical anesthesia is usually adopted before tracheal intubation. Specifically, local anesthetics such as lidocaine are mainly used for infiltration anesthesia of nasal mucosa and throat surface, which can function rapidly, block partial receptor reflexes under the mucosae of nasal cavity, throat and airway and inhibit the afference of nerve impulse, thus relieving sympathetic-adrenal medulla axis reaction in patients^16^-^19^.

Nevertheless, favorable anesthetic effect cannot be achieved in some patients receiving tracheal intubation via simple topical anesthesia, and such patients are vulnerable to blood pressure elevation, heart rate increase and other stress responses as well as coughing, restlessness and other adverse reactions. In view of this situation, right stellate ganglion block is proposed clinically, in which lidocaine injection is mainly injected into the sympathetic ganglion of the neck and chest to block excitability transmission of sympathetic nerve.

Additionally, it can act on the hypothalamic-pituitary-adrenal axis system, repress the release of norepinephrine and neuropeptide Y, thereby decreasing the tension of stellate ganglion, relieving the hyperfunctional state, regulating the autonomic nerve and avoiding tension imbalance between sympathetic nerve and vagus nerve during tracheal intubation of patients^16^. Furthermore, as the right vagus nerve is close to the right stellate ganglion, lidocaine is able to efficiently block the sympathetic nerve and vagus nerve. In this research, it was found that (1) the excellent and good rate of anesthesia was 98.33% in observation group, which was higher than that (86.67%) in control group (P<0.05). Compared with control group, observation group exhibited reduced systolic pressure, diastolic pressure and heart rate (P<0.05). Besides, the low-frequency power, ratio of low-frequency power to high-frequency power and heart rate variability index were higher in observation group than those in control group (P<0.05).

Observation group had a lower incidence rate of adverse reactions of tracheal intubation (P<0.05), a higher success rate of tracheal intubation (P<0.05) and less time consumption of tracheal intubation (P<0.05) than control group. All these results suggest that the right stellate ganglion block based on topical anesthesia can substantially improve the anesthetic effect before tracheal intubation, maintain stable vital signs and minimize such adverse reactions as coughing and restlessness, so as to ensure successful tracheal intubation. The differences in recovery time of spontaneous respiration, time to eye opening and response recovery time to command after operation were not significant between observation group and control group (P>0.05), and no significant changes in MMSE and MoCA scores were discovered after operation compared with those before operation (P>0.05), implying that the right stellate ganglion block combined with topical anesthesia does not delay the postoperative resuscitation of patients or prominently impair the cognitive function.

In conclusion, right stellate ganglion block combined with topical anesthesia can effectively improve the anesthetic effect on awake patients during tracheal intubation, relieve the fluctuations of vital signs, maintain stable circulatory function and reduce the incidence rate of adverse reactions. This method is conducive to increasing the success rate of tracheal intubation and decreasing the time consumption of intubation while hardly affecting the postoperative resuscitation and cognitive function of patients. Regardless, this study still has limitations. First, the sample size was small. Second, follow-up was not conducted. Further multicenter studies with larger sample sizes and long-term follow-up are ongoing in our group to verify the conclusion of this study.
